# Dynamic stability analysis of a fractional calculus-based colon cancer model with therapeutic interventions

**DOI:** 10.1038/s41598-025-31328-z

**Published:** 2025-12-10

**Authors:** Ruilei Tai, Zhaoyang Chen, Yanling Zhao, Ruiqing Shi

**Affiliations:** 1 Laboratory Animal Center, Shanxi Key Laboratory of Experimental Animal Science and Animal Model of Human Disease, Shanxi Medical University, Taiyuan, 030001 China; 2 Shanxi Key Laboratory of Cryptography and Data Security, School of Mathematical Sciences, Shanxi Normal University, Taiyuan, 030031 China

**Keywords:** Fractional order, Colon cancer model, Equilibriums, Stability, Treatment strategies, Cancer, Computational biology and bioinformatics, Immunology

## Abstract

Colon cancer is a high-risk malignant tumor worldwide. To address the limited efficacy and overlapping toxicity of current chemoimmunotherapy, this study establishes a fractional-order colon cancer model with a core architecture capturing the probiotics-$\hbox {CD4}^{+}$T cells-tumor cells axis. The primary theoretical contributions include: rigorous proof of solution existence and uniqueness through fixed-point theory. Analytical derivation of equilibrium stability conditions using the Routh-Hurwitz criterion. Numerical simulations systematically quantify how the fractional parameter $\alpha$ governs system dynamics and memory effects, while identifying critical drug dosage thresholds for tumor clearance. These findings provide a mathematical foundation for optimizing probiotic-based combination therapies.

## Introduction

Globally, colorectal cancer (CRC) ranks as the third most prevalent cancer and stands as the second primary reason for cancer-associated mortalities, exerting a grave menace to human health^1^. Currently, combination therapy involving chemotherapeutic agents and immune checkpoint inhibitors (ICIs) has demonstrated synergistic antitumor effects: chemotherapeutic agents directly kill tumor cells to release antigens, activating systemic immune responses to enhance ICI efficacy. Meanwhile, ICIs block immunosuppressive pathways, activate T cell-mediated antitumor responses, and remodel the tumor microenvironment (TME) to downregulate chemotherapy resistance proteins, thereby bidirectionally improving treatment sensitivity. However, this combination strategy faces multiple challenges. Overlapping toxicities-such as chemotherapy-induced myelosuppression, gastrointestinal toxicity, and immune-related adverse events (e.g., colitis, hepatitis)-significantly reduce patients’ quality of life. Furthermore, the therapeutic benefit remains limited for 85% of patients with pMMR/MSS-type CRC, failing to overcome the “cold tumor” barrier^2^. Emerging evidence suggests that specific probiotics can achieve dual modulation: reshaping the immune microenvironment to promote $\hbox {CD4}^{+}$/CD$8^{+}$ T cell infiltration and reverse immunosuppression, thereby enhancing chemoimmunotherapy efficacy, and regulating gut barrier integrity and microbiota homeostasis to alleviate chemotherapy-induced mucosal damage and immune toxicity, offering a novel direction to address current limitations.

Probiotics are scientifically validated live microorganisms that confer health benefits primarily through gut colonization, microbiota modulation, and host immune regulation, exhibiting multifaceted roles in CRC prevention and treatment. In vitro studies show that *Lactobacillus rhamnosus* supernatant suppresses proliferation and induces apoptosis in CRC cell lines (HT29, HCT-116, Caco-2), arresting cells in the G0/G1 phase^3^. Experimental models demonstrate that probiotics exert direct antitumor effects and synergize with conventional therapies by modulating key pathways and the gut microbiome. In AOM-DSS-induced mouse models, prophylactic administration of *Bifidobacterium lactis* with prebiotics alleviates weight loss, colon shortening, and precancerous lesion development^4^. Beyond single strains, probiotic combinations (e.g., *Clostridium butyricum + Akkermansia muciniphila*) demonstrate synergistic efficacy, improving anti-PD-L1 responses and survival in colitis-associated CRC mice by suppressing inflammation and oxidative stress^5^. This is supported by clinical studies, where probiotic administration significantly mitigates postoperative complications and chemotherapy-related toxicity, while improving patient survival and quality of life^6,7^.

Mathematical modeling provides a powerful quantitative framework to decipher the complex, non-linear dynamics of tumor-immune interactions. While integer-order differential equations have been widely employed, fractional-order calculus offers a superior framework for biological systems exhibiting memory and hereditary properties^8^. The kernel $(t-\tau )^{(\alpha -1)}$ in the definition of the fractional integral/derivative assigns a weight to past states, with $\alpha$ serving as a parameter that modulates the strength of this memory: values of $\alpha$ less than one indicate a strong dependence on history, a phenomenon often observed in complex biological processes^9^. The non-local kernel of fractional operators, such as the Caputo derivative used in this study, inherently captures the temporal memory effects prevalent in immune cell activation, proliferation, and drug pharmacokinetics/pharmacodynamics^10–12^. Owing to these advantages, fractional calculus has been extensively utilized across diverse fields including physics^13^, chemistry^14^, financial science^15^, engineering^16^ and cybersecurity^17^. Its power in modeling complex biological phenomena is also demonstrated in other medical fields, such as the analysis of Hyers-Ulam stability for delayed systems relevant to infectious diseases^18^, leveraging the same fractional calculus tools that provide a robust framework for advanced neural network models^19^. Notably, its application in oncology is gaining significant traction, as seen in models evaluating the efficacy of therapies like ABT-737 in reproductive cancer apoptosis using advanced fractional operators^20^.

Building upon the broad utility of fractional calculus, several studies have pioneered its application in modeling colon cancer. Manadi et.al^21^ developed a fractional-order model to investigate the interactions between tumor cells and macrophage cells, while Yıldız et al.^22^ applied a similar approach to explore the role of fat cells. Concurrently, research has incorporated therapeutic interventions: a fractal-fractional model^23^ confirms the potent efficacy of a single-dose anti-PD-L1 immunotherapy in significantly clearing tumor cells, and another simulated cetuximab treatment, capturing its dynamic impact on the tumor microenvironment^24^. Chemo-immunotherapy combination, by enhancing IL-2 levels and employing the mAb Cetuximab, potentiates the anti-tumor activity of immune effector cells (such as $\hbox {CD8}^{+}$T cells and NK cells), leading to effective tumor control^25^. Biomedical research on the impact of probiotics on colorectal cancer has revealed multiple anti-tumor mechanisms, particularly through the modulation of host immune responses, which provides a critical biological foundation of hypotheses and interactive relationships for constructing corresponding mathematical models. However, studies that incorporate probiotics as an external input or systemic variable-capable of positively regulating immune cell cytotoxicity or negatively regulating tumor cell proliferation-within a mathematical modeling framework remain relatively scarce. The Microbial Tumor Evolution Equation (MTEE), proposed by Sakib et al.^26^, offers a mathematical framework for quantifying interactions between microbial therapies (including probiotics) and tumors, with simulations predicting that probiotic intervention can suppress tumor growth and enhance the efficacy of immunotherapy. Despite these advances, a significant research gap persists: no existing model integrates probiotics as a dynamic intervention variable while specifically focusing on the critical coordinating role of $\hbox {CD4}^{+}$T cells in this process.

This gap is bridged through a strategic re-focusing onto the immunoregulatory hub of $\hbox {CD4}^{+}$T cells, coupled with the explicit integration of probiotic intervention as a novel dynamic variable alongside chemo-immunotherapy-a dual emphasis that provides fresh mechanistic insights into optimizing combination regimens. Inspired by prior research^22,27,28^, this paper presents a fractional-order mathematical model of colon cancer. The model describes the interactions among tumor cells *T*(*t*), macrophages *M*(*t*), dendritic cells *G*(*t*), $\hbox {CD4}^{+}$T helper cells *C*(*t*), and probiotic drug *P*(*t*).

1 $$\begin{aligned} \left\{ \begin{array}{lll} \displaystyle ^{C}_{0}D_{t}^{\alpha }T(t)=g_{1}T(t)\left( 1 -\frac{T(t)}{s_{1}}\right) -\beta _{1}\frac{T(t)M(t)}{s_{2}+T(t)}-\omega \frac{T(t)C(t)}{s_{4}+T(t)}-u_{3}(t)T(t),\\ \displaystyle ^{C}_{0}D_{t}^{\alpha }M(t)=g_{2}\frac{T(t)}{s_{2}+T(t)}-\beta _{2}M(t)-d_{1}M(t),\\ \displaystyle ^{C}_{0}D_{t}^{\alpha }G(t)=r_{1}+g_{3}\frac{T(t)}{s_{3}+T(t)}-\beta _{3}G(t)-d_{2}G(t),\\ \displaystyle ^{C}_{0}D_{t}^{\alpha }C(t)=r_{2}+g_{4}G(t)-\beta _{4}C(t)-d_{3}C(t)+\frac{\delta P(t)C(t)}{b+P(t)}+u_{2}(t)C(t),\\ \displaystyle ^{C}_{0}D_{t}^{\alpha }P(t)=u_{1}(t)-\vartheta P(t), \end{array}\right. \end{aligned}$$

with initial conditions

$$\begin{aligned} T(0)=T_{0},\quad M(0)=M_{0},\quad G(0)=G_{0},\quad C(0)=C_{0},\quad P(0)=P_{0}. \end{aligned}$$

In order to make the time dimensions on both sides of the equation consistent, we adopt the method in^29^.

2 $$\begin{aligned} \left\{ \begin{array}{lll} \frac{1}{\sigma ^{1-\alpha }}\displaystyle ^{C}_{0}D_{t}^{\alpha }T(t)=\displaystyle g_{1}T(t)\left( 1-\frac{T(t)}{s_{1}}\right) -\beta _{1}\frac{T(t)M(t)}{s_{2}+T(t)}-\omega \frac{T(t)C(t)}{s_{4}+T(t)}-u_{3}(t)T(t),\\ \frac{1}{\sigma ^{1-\alpha }}\displaystyle ^{C}_{0}D_{t}^{\alpha }M(t)=\displaystyle g_{2}\frac{T(t)}{s_{2}+T(t)}-\beta _{2}M(t)-d_{1}M(t),\\ \frac{1}{\sigma ^{1-\alpha }}\displaystyle ^{C}_{0}D_{t}^{\alpha }G(t)=\displaystyle r_{1}+g_{3}\frac{T(t)}{s_{3}+T(t)}-\beta _{3}G(t)-d_{2}G(t),\\ \frac{1}{\sigma ^{1-\alpha }}\displaystyle ^{C}_{0}D_{t}^{\alpha }C(t)=\displaystyle r_{2}+g_{4}G(t)-\beta _{4}C(t)-d_{3}C(t)+\frac{\delta P(t)C(t)}{b+P(t)}+u_{2}(t)C(t),\\ \frac{1}{\sigma ^{1-\alpha }}\displaystyle ^{C}_{0}D_{t}^{\alpha }P(t)=\displaystyle u_{1}(t)-\vartheta P(t). \end{array}\right. \end{aligned}$$

The specific biological meanings of all parameters are listed in Table 1.

The structure of this paper is organized as follows: Sect. "The existence and uniqueness of solutions of system (2)" proves the existence and uniqueness of the solution to system (2). Section "Analysis of the equilibriums of system (2)" investigates the stability of the equilibriums of system (2). Section "Examples and numerical simulations" presents and analyzes numerical simulations. and the final section summarizes and discusses the research, outlines future research directions, and provides insights for the development of related fields.

**Table 1:** The biological significance of the parameters of system $(2)$.

Parameters	Descriptions	Values	References
${g_{1}}$	Intrinsic growth rate of tumor cells	[0.1, 0.9]	^28^
${s_{1}}$	Carrying capacity of the environment for tumor cells	1000	^28^
${s_{2}}$	Half-saturation constant for macrophages	50	^28^
${s_{3}}$	Half-saturation constant for dendritic cells	50	^28^
${s_{4}}$	Half-saturation constant for $\hbox {CD4}^{+}$T cells	50	^28^
${r_{1}}$	The production rate of Dendritic cells	0.3	^28^
${r_{2}}$	The production rate of $\hbox {CD4}^{+}$T cells	0.7	^28^
${\beta _{1}}$	Rate at which tumor cells are killed by macrophages	0.1	^28^
${\omega }$	Rate at which tumor cells are killed by $\hbox {CD4}^{+}$T cells	0.3	^22^
${g_{2}}$	Recruitment rate of macrophages by tumor cells	[0.2, 0.9]	^28^
${\beta _{2}}$	Natural death rate of macrophages	0.5	^28^
${g_{3}}$	Recruitment rate of dendritic cells by tumor cells	[0.2, 0.9]	^28^
${\beta _{3}}$	Natural death rate of dendritic cells	0.4	^28^
${g_{4}}$	Activation rate of $\hbox {CD4}^{+}$T cells by dendritic cells	[0.1, 0.9]	^28^
${\beta _{4}}$	Natural death rate of $\hbox {CD4}^{+}$T cells	0.7	^28^
${d_{1}}$	Represents the death rate of macrophages that		
	lose their activity under the effect of chemotherapy	0.008	^22^
${d_{2}}$	Represents the death rate of dendritic cells that		
	lose their activity under the effect of chemotherapy	0.0008	^22^
${d_{3}}$	Represents the death rate of $\hbox {CD4}^{+}$T helper cells that		
	lose their activity under the effect of chemotherapy	0.0001	^22^
${\vartheta }$	Decay rate of *P*	1	^22^
${\delta }$	Recruitment rate of $\hbox {CD4}^{+}$T helper cells by *P*	[0.15, 0.60]	^22^
*b*	Half-saturation constant for a probiotic drug	0.25	^22^
${u_{1}}$	Represents the dosage of the probiotic drug	–	–
${u_{2}}$	Represents the dosage of the immunotherapeutic drug	–	–
${u_{3}}$	Represents the dosage of the chemotherapy drug	–	–

## The existence and uniqueness of solutions of system (2)

Firstly we define the following kernels:

3 $$\begin{aligned} \begin{aligned} \mathfrak {K_{1}}(t,T)&=\displaystyle g_{1}T(t)\left( 1-\frac{T(t)}{s_{1}}\right) -\beta _{1}\frac{T(t)M(t)}{s_{2}+T(t)}-\omega \frac{T(t)C(t)}{s_{4}+T(t)}-u_{3}(t)T(t),\\ \mathfrak {K_{2}}(t,M)&=\displaystyle g_{2}\frac{T(t)}{s_{2}+T(t)}-\beta _{2}M(t)-d_{1}M(t),\\ \mathfrak {K_{3}}(t,G)&=\displaystyle r_{1}+g_{3}\frac{T(t)}{s_{3}+T(t)}-\beta _{3}G(t)-d_{2}G(t),\\ \mathfrak {K_{4}}(t,C)&=\displaystyle r_{2}+g_{4}G(t)-\beta _{4}C(t)-d_{3}C(t)+\frac{\delta P(t)C(t)}{b+P(t)}+u_{2}(t)C(t),\\ \mathfrak {K_{5}}(t,P)&=\displaystyle u_{1}(t)-\vartheta P(t). \end{aligned} \end{aligned}$$

Based on the above kernel functions, system (2) can be expressed as:

4 $$\begin{aligned} \begin{aligned} \displaystyle \frac{1}{\sigma ^{1-\alpha }}\displaystyle ^{C}_{0}D_{t}^{\alpha }T(t)&=\mathfrak {K_{1}}(t,T),\\ \displaystyle \frac{1}{\sigma ^{1-\alpha }}\displaystyle ^{C}_{0}D_{t}^{\alpha }M(t)&=\mathfrak {K_{2}}(t,M),\\ \displaystyle \frac{1}{\sigma ^{1-\alpha }}\displaystyle ^{C}_{0}D_{t}^{\alpha }G(t)&=\mathfrak {K_{3}}(t,G),\\ \displaystyle \frac{1}{\sigma ^{1-\alpha }}\displaystyle ^{C}_{0}D_{t}^{\alpha }C(t)&=\mathfrak {K_{4}}(t,C),\\ \displaystyle \frac{1}{\sigma ^{1-\alpha }}\displaystyle ^{C}_{0}D_{t}^{\alpha }P(t)&=\mathfrak {K_{5}}(t,P). \end{aligned} \end{aligned}$$

By utilizing the Lemma 1 in^29^ and according to the initial value conditions, we perform the Riemann-Liouville fractional integral on both sides of Eq. (4), and finally obtain

5 $$\begin{aligned} \begin{array}{lll} T(t)-T_{0}=\frac{\sigma ^{1-\alpha }}{\Gamma (\alpha )}\int _{0}^{t}\mathfrak {K_{1}}(t,T)(t-\tau )^{\alpha -1}\textrm{d}\tau ,\\ M(t)-M_{0}=\frac{\sigma ^{1-\alpha }}{\Gamma (\alpha )}\int _{0}^{t}\mathfrak {K_{2}}(t,M)(t-\tau )^{\alpha -1}\textrm{d}\tau ,\\ G(t)-G_{0}=\frac{\sigma ^{1-\alpha }}{\Gamma (\alpha )}\int _{0}^{t}\mathfrak {K_{3}}(t,G)(t-\tau )^{\alpha -1}\textrm{d}\tau ,\\ C(t)-C_{0}=\frac{\sigma ^{1-\alpha }}{\Gamma (\alpha )}\int _{0}^{t}\mathfrak {K_{4}}(t,C)(t-\tau )^{\alpha -1}\textrm{d}\tau ,\\ P(t)-P_{0}=\frac{\sigma ^{1-\alpha }}{\Gamma (\alpha )}\int _{0}^{t}\mathfrak {K_{5}}(t,P)(t-\tau )^{\alpha -1}\textrm{d}\tau . \end{array} \end{aligned}$$

To verify that the kernel functions in Eq.(3) satisfies the Lipschitz condition, the following assumptions are now introduced:

$\mathfrak {B}$: For the continuous functions *T*(*t*), *M*(*t*), *G*(*t*), *C*(*t*), *P*(*t*) belong to *L*[0, *a*], there exist constants $b_{i}\in N$, $i=1,2,3,4,5$, such that the following hold:

6 $$\begin{aligned} \Vert T\Vert<b_{1},\quad \Vert M\Vert<b_{2},\quad \Vert G\Vert<b_{3},\quad \Vert C\Vert<b_{4},\quad \Vert P\Vert <b_{5}. \end{aligned}$$

Now, we put forward the following result.

### Theorem 1

The kernels $\mathfrak {K_{1}}(t,T)$, $\mathfrak {K_{2}}(t,M)$, $\mathfrak {K_{3}}(t,G)$, $\mathfrak {K_{4}}(t,C)$, and $\mathfrak {K_{5}}(t,P)$ satisfy the Lipschitz condition, if hypothesis $\mathfrak {B}$ holds and the following inequalities are satisfied (the symbol *a* is defined as a constant):

7 $$\begin{aligned} \begin{aligned} L_{1}&=g_{1}+u_{3}+\frac{2b_{1}g_{1}}{s_{1}}+\frac{\beta _{1}b_{2}}{s_{2}}+\frac{\omega b_{4}}{s_{4}}<a,\\ L_{2}&=\beta _{2}+d_{1}<a,\quad L_{3}=\beta _{3}+d_{2}<a,\\ L_{4}&=\beta _{4}+d_{3}+\delta +u_{2}<a,\quad L_{5}=\vartheta <a. \end{aligned} \end{aligned}$$

### Proof

Based on the definition of $\mathfrak {K_{1}}(t,T)$ in Eq.(3) and combined with hypothesis $\mathfrak {B}$, by conducting derivations for *T* and $T^{*}$, we can obtain:

8 $$\begin{aligned} \begin{aligned} \Vert \mathfrak {K_{1}}(t,T)-\mathfrak {K_{1}}(t,T^{*})\Vert =&\Vert g_{1}(T-T^{*})-\frac{g_{1}}{s_{1}}(T^{2}-(T^{*})^{2}) -\beta _{1}Ms_{2}\frac{T-T^{*}}{(s_{2}+T)(s_{2}+T^{*})}\\&-\omega s_{4}C\frac{T-T^{*}}{(s_{4}+T)(s_{4}+T^{*})}-u_{3}(T-T^{*})\Vert \\ \le&g_{1}\Vert T-T^{*}\Vert +\frac{g_{1}}{s_{1}}\Vert T+T^{*}\Vert \Vert T-T^{*}\Vert +\beta _{1}s_{2}\Vert M\Vert \left\| \frac{T-T^{*}}{(s_{2}+T)(s_{2}+T^{*})}\right\| \\&+\omega s_{4}\Vert C\Vert \left\| \frac{T-T^{*}}{(s_{4}+T)(s_{4}+T^{*})}\right\| +u_{3}\Vert T-T^{*}\Vert \\ \le&\left( g_{1}+u_{3}+\frac{2b_{1}g_{1}}{s_{1}}+\frac{\beta _{1}b_{2}}{s_{2}}+\frac{\omega b_{4}}{s_{4}}\right) \Vert T-T^{*}\Vert \\ =&L_{1}\Vert T-T^{*}\Vert . \end{aligned} \end{aligned}$$

It can be seen from this that the kernel $\mathfrak {K_{1}}(t,T)$ satisfies the Lipschitz condition. For the kernel $\mathfrak {K_{2}}(t,M)$ in Eq.(3), we obtain the following conclusions regarding *M* and $M^{*}$:

9 $$\begin{aligned} \begin{aligned} \Vert \mathfrak {K_{2}}(t,M)-\mathfrak {K_{2}}(t,M^{*})\Vert =&\Vert -\beta _{2}(M-M^{*})-d_{1}(M-M^{*})\Vert \\ \le&(\beta _{2}+d_{1})\Vert M-M^{*}\Vert \\ =&L_{2}\Vert M-M^{*}\Vert . \end{aligned} \end{aligned}$$

This indicates that the kernel $\mathfrak {K_{2}}(t,M)$ satisfies the Lipschitz condition. In terms of the continuous functions *G* and $G^{*}$, we have derived the following results:

10 $$\begin{aligned} \begin{aligned} \Vert \mathfrak {K_{3}}(t,G)-\mathfrak {K_{3}}(t,G^{*})\Vert =&\Vert -\beta _{3}(G-G^{*})-d_{2}(G-G^{*})\Vert \\ \le&(\beta _{3}+d_{2})\Vert (G-G^{*})\Vert \\ =&L_{3}\Vert G-G^{*}\Vert . \end{aligned} \end{aligned}$$

It can be concluded from this that the kernel $\mathfrak {K_{3}}(t,G)$ satisfies the Lipschitz condition. Now, we set out to prove that the kernel $\mathfrak {K_{4}}(t,C)$ in Eq.(3) also satisfies this condition. Regarding the continuous functions *C* and $C^{*}$, we obtain the following conclusions:

11 $$\begin{aligned} \begin{aligned} \Vert \mathfrak {K_{4}}(t,C)-\mathfrak {K_{4}}(t,C^{*})\Vert =&\Vert -\beta _{4}(C-C^{*})-d_{3}(C-C^{*})+\frac{\delta P}{b+P}(C-C^{*})+u_{2}(C-C^{*})\Vert \\ \le&(\beta _{4}+d_{3}+\delta +u_{2})\Vert C-C^{*}\Vert \\ =&L_{4}\Vert C-C^{*}\Vert . \end{aligned} \end{aligned}$$

Therefore, $\mathfrak {K_{4}}(t,C)$ satisfies the Lipschitz condition. It is obvious that *P* and $P^{*}$ are continuous functions, and:

12 $$\begin{aligned} \begin{aligned} \Vert \mathfrak {K_{5}}(t,P)-\mathfrak {K_{5}}(t,P^{*})\Vert =&\Vert -\vartheta (P-P^{*})\Vert \\ \le&\vartheta \Vert (P-P^{*})\Vert \\ =&L_{5}\Vert (P-P^{*})\Vert . \end{aligned} \end{aligned}$$

Therefore the kernel $\mathfrak {K_{5}}(t,P)$ satisfies the Lipschitz condition, and thus the proof is completed. $\square$

Now, the differences between two successive components of *T*(*t*), *M*(*t*), *G*(*t*), *C*(*t*) and *P*(*t*) are defined as follows:

13 $$\begin{aligned} \begin{array}{llll} \displaystyle \Psi _{n}^{1}(t)& =T_{n}(t)-T_{n-1}(t)& =\displaystyle \frac{\sigma ^{1-\alpha }}{\Gamma (\alpha )}\int _{0}^{t}(\mathfrak {K_{1}}(t,T_{n-1}) -\mathfrak {K_{1}}(t,T_{n-2}))(t-\tau )^{\alpha -1}d\tau ,\\ \displaystyle \Psi _{n}^{2}(t)& =M_{n}(t)-M_{n-1}(t)& =\displaystyle \frac{\sigma ^{1-\alpha }}{\Gamma (\alpha )}\int _{0}^{t}(\mathfrak {K_{2}}(t,M_{n-1}) -\mathfrak {K_{2}}(t,M_{n-2}))(t-\tau )^{\alpha -1}d\tau ,\\ \displaystyle \Psi _{n}^{3}(t)& =G_{n}(t)-G_{n-1}(t)& =\displaystyle \frac{\sigma ^{1-\alpha }}{\Gamma (\alpha )}\int _{0}^{t}(\mathfrak {K_{3}}(t,G_{n-1}) -\mathfrak {K_{3}}(t,G_{n-2}))(t-\tau )^{\alpha -1}d\tau ,\\ \displaystyle \Psi _{n}^{4}(t)& =C_{n}(t)-C_{n-1}(t)& =\displaystyle \frac{\sigma ^{1-\alpha }}{\Gamma (\alpha )}\int _{0}^{t}(\mathfrak {K_{4}}(t,C_{n-1}) -\mathfrak {K_{4}}(t,C_{n-2}))(t-\tau )^{\alpha -1}d\tau ,\\ \displaystyle \Psi _{n}^{5}(t)& =P_{n}(t)-P_{n-1}(t)& =\displaystyle \frac{\sigma ^{1-\alpha }}{\Gamma (\alpha )}\int _{0}^{t}(\mathfrak {K_{5}}(t,P_{n-1}) -\mathfrak {K_{5}}(t,P_{n-2}))(t-\tau )^{\alpha -1}d\tau , \end{array} \end{aligned}$$

with the initial conditions. Therefore we obtain that:

14 $$\begin{aligned} \begin{aligned} T_{n}(t)&=\sum _{i=0}^{n}\Psi _{i}^{1}(t),\quad M_{n}(t)=\sum _{i=0}^{n}\Psi _{i}^{2}(t),\quad G_{n}(t)=\sum _{i=0}^{n}\Psi _{i}^{3}(t),\\ C_{n}(t)&=\sum _{i=0}^{n}\Psi _{i}^{4}(t),\quad P_{n}(t)=\sum _{i=0}^{n}\Psi _{i}^{5}(t). \end{aligned} \end{aligned}$$

Based on the norm property of Eq.(13) and combined with the Lipschitz condition Eq.(8), we can derive:

15 $$\begin{aligned} \begin{aligned} \displaystyle \Vert \Psi _{n}^{1}(t)\Vert&=\Vert T_{n}(t)-T_{n-1}(t)\Vert \\ \displaystyle&=\left\| \frac{\sigma ^{1-\alpha }}{\Gamma (\alpha )}\int _{0}^{t}(\mathfrak {K_{1}}(t,T_{n-1}) \displaystyle -\mathfrak {K_{1}}(t,T_{n-2}))(t-\tau )^{\alpha -1}d\tau \right\| \\&\le \frac{\sigma ^{1-\alpha }}{\Gamma (\alpha )}\int _{0}^{t}\Vert (\mathfrak {K_{1}}(t,T_{n-1}) \displaystyle -\mathfrak {K_{1}}(t,T_{n-2}))(t-\tau )^{\alpha -1}\Vert d\tau \\&\le \frac{\sigma ^{1-\alpha }}{\Gamma (\alpha )}L_{1}\int _{0}^{t}\Vert \Psi _{n-1}^{1}(t)\Vert d\tau . \end{aligned} \end{aligned}$$

Similarly, the following conclusions can be drawn:

16 $$\begin{aligned} \begin{array}{rlc} \displaystyle \Vert \Psi _{n}^{2}(t)\Vert \le \frac{\sigma ^{1-\alpha }}{\Gamma (\alpha )}L_{2}\int _{0}^{t}\Vert \Psi _{n-1}^{2}(t)\Vert d\tau ,\\ \displaystyle \Vert \Psi _{n}^{3}(t)\Vert \le \frac{\sigma ^{1-\alpha }}{\Gamma (\alpha )}L_{3}\int _{0}^{t}\Vert \Psi _{n-1}^{3}(t)\Vert d\tau ,\\ \displaystyle \Vert \Psi _{n}^{4}(t)\Vert \le \frac{\sigma ^{1-\alpha }}{\Gamma (\alpha )}L_{4}\int _{0}^{t}\Vert \Psi _{n-1}^{4}(t)\Vert d\tau ,\\ \displaystyle \Vert \Psi _{n}^{5}(t)\Vert \le \frac{\sigma ^{1-\alpha }}{\Gamma (\alpha )}L_{5}\int _{0}^{t}\Vert \Psi _{n-1}^{5}(t)\Vert d\tau . \end{array} \end{aligned}$$

Next, we will expound on the following theorem.

### Theorem 2

If the inequalities in Eqs.(6) and (7), as well as the inequality

$$\begin{aligned} L^{*}=\mathrm {\max }\ \{L_{i}, i=1,2,3,4,5\}<a \end{aligned}$$

all hold, then there exists a solution for system (2).

### Proof

Firstly, we define the following functions.

17 $$\begin{aligned} \begin{aligned} \mathfrak {K_{1,n}}(t)&=T_{n+1}(t)-T(t),\quad \mathfrak {K_{2,n}}(t)=M_{n+1}(t)-M(t),\quad \mathfrak {K_{3,n}}(t)=G_{n+1}(t)-G(t),\\ \mathfrak {K_{4,n}}(t)&=C_{n+1}(t)-C(t),\quad \mathfrak {K_{5,n}}(t)=P_{n+1}(t)-P(t). \end{aligned} \end{aligned}$$

By using Eq.(15) to derive the function $\mathfrak {K_{1,n}}(t)$, the following conclusions can be obtained:

18 $$\begin{aligned} \begin{aligned} \Vert \mathfrak {K_{1,n}}(t)\Vert =&\Vert T_{n+1}(t)-T(t)\Vert \\ \displaystyle \le&\left( \frac{\sigma ^{1-\alpha }}{\Gamma (\alpha )}\right) ^{n}(L^{*})^{n}\Vert T_{1}(t)-T(t)\Vert , \end{aligned} \end{aligned}$$

which means $\mathfrak {K_{1,n}}(t)\rightarrow 0$ as $n\rightarrow \infty$. Similarly, it can be proved that when $n\rightarrow \infty$, for the cases where $\mathfrak {K_{i,n}}(t)\rightarrow 0$ ($i=2,3,4,5$), which completes the entire proof process. $\square$

Next, we will prove the uniqueness of the solution of system (2).

### Theorem 3

If the inequalities in Eq.(6) and (7), as well as

19 $$\begin{aligned} \begin{aligned} 1-\frac{\sigma ^{1-\alpha }}{\Gamma (\alpha )}L_{i}\ge 0 ,\quad i = 1, 2, 3, 4, 5 \end{aligned} \end{aligned}$$

hold, then system (2) has a unique solution.

### Proof

Suppose that there exists another set of solutions $(T^{*}, M^{*}, G^{*}, C^{*}, P^{*})$ for system (2). It can be seen from Eq. (5) that $T^{*}(t)$ satisfies the following integral equation:

20 $$\begin{aligned} \begin{aligned} T^{*}=T(0)+\frac{\sigma ^{1-\alpha }}{\Gamma (\alpha )}\int _{0}^{t}\mathfrak {K_{1}}(t,T^{*})(t-\tau )^{\alpha -1}\textrm{d}\tau . \end{aligned} \end{aligned}$$

Therefore, based on the calculation results of Eq.(8), we can derive that:

21 $$\begin{aligned} \begin{aligned} \Vert T_{1}(t)-T^{*}(t)\Vert&\le \frac{\sigma ^{1-\alpha }}{\Gamma (\alpha )}\int _{0}^{t}\Vert (\mathfrak {K_{1}}(t,T_{1})-\mathfrak {K_{1}}(t,T^{*}))(t-\tau )^{\alpha -1}\Vert \textrm{d}\tau \\&\le \frac{\sigma ^{1-\alpha }}{\Gamma (\alpha )}L_{1}\Vert T_{1}(t)-T^{*}(t)\Vert . \end{aligned} \end{aligned}$$

This gives:

22 $$\begin{aligned} \begin{aligned} \left( 1-\frac{\sigma ^{1-\alpha }}{\Gamma (\alpha )}L_{1}\right) \Vert T_{1}(t)-T^{*}(t)\Vert \le 0. \end{aligned} \end{aligned}$$

By utilizing this result and combining it with the conditions in Eq. (19), we have derived that $T_{1}(t)=T^{*}(t)$. Similarly, through a similar derivation process, the uniqueness of solutions can be proven. $\square$

## Analysis of the equilibriums of system(2)

First of all, we focus on exploring the characteristics of the equilibrium of this model. For the given model, its core non-trivial condition is that when the number of tumor cells reaches zero, the state of the system at this time is what we refer to as the disease-free state. This state can be achieved by setting the number of tumor cells in the given model to zero. It has been verified that the tumor-free equilibrium $E_1$ of system (2) can be expressed as $E_1^{*}=(0, 0, G_{1}^{*}, C_{1}^{*}, P_{1}^{*})$, where,

23 $$\begin{aligned} G_{1}^{*}=\displaystyle \frac{r_{1}}{\beta _{3}+d_{2}},\quad C_{1}^{*}=\displaystyle \frac{[r_{2(\beta _{3}+d_{2})+g_{4}r_{4}}](\vartheta b+u_{1})}{[(\vartheta b+u_{1})(\beta _{4}+d_{3}-u_{2})-\delta u_{1})](\beta _{3}+d_{2})},\quad P_{1}^{*}=\displaystyle \frac{u_{1}}{\vartheta }. \end{aligned}$$

Next, we will further explore the coexistence equilibrium.

24 $$\begin{aligned} \left\{ \begin{array}{lll} \displaystyle g_{1}T\left( 1-\frac{T}{s_{1}}\right) -\beta _{1}\frac{TM}{s_{2}+T}-\omega \frac{TC}{s_{4}+T}-u_{3}T=0,\\ \displaystyle g_{2}\frac{T}{s_{2}+T}-\beta _{2}M-d_{1}M=0,\\ \displaystyle r_{1}+g_{3}\frac{T}{s_{3}+T}-\beta _{3}G-d_{2}G=0,\\ \displaystyle r_{2}+g_{4}G-\beta _{4}C-d_{3}C+\frac{\delta PC}{b+P}+u_{2}C=0,\\ \displaystyle u_{1}-\vartheta P=0. \end{array}\right. \end{aligned}$$

So, the coexistence equilibrium $E_2^{*}=(T_{2}^{*}, M_{2}^{*}, G_{2}^{*}, C_{2}^{*}, P_{2}^{*})$, where,

25 $$\begin{aligned} \begin{array}{lll} M_{2}^{*}& =\displaystyle \frac{g_{2}T_{2}^{*}}{(\beta _{2}+d_{1})(s_{2}+T_{2}^{*})},\quad G_{2}^{*}=\displaystyle \frac{(\beta _{4}+d_{3}-u_{2}-\frac{\delta P_{2}^{*}}{b+P_{2}^{*}})C_{2}^{*}-r_{2}}{g_{4}},\\ C_{2}^{*}& =\displaystyle \frac{r_{1}+\frac{g_{3}T_{2}^{*}}{s_{3}+T_{2}^{*}}}{\beta _{3}+d_{2}},\quad P_{2}^{*}=\displaystyle \frac{u_{1}}{\vartheta }, \end{array} \end{aligned}$$

and $T_{2}^{*}$ is the positive solution of the following equation,

26 $$\begin{aligned} \displaystyle g_{1}\left( 1-\frac{T_{2}}{s_{1}}\right) -\frac{\beta _{1}g_{2}T_{2}}{(\beta _{2}+d_{1})(s_{2}+T_{2})^{2}}-\frac{\omega r_{1}(s_{3}+T_{2})+\omega g_{3}T_{2}}{(\beta _{3}+d_{2})(s_{3}+T_{2})(s_{4}+T_{2})}-u_{3}=0. \end{aligned}$$

Next, we will investigate the stability of the equilibriums.

(i) The Jacobian matrix of system (2) at equilibrium $E_{1}^{*}$ is

27 $$\begin{aligned} J(E_{1}^{*})= \left( \begin{array}{cccccc} \displaystyle g_{1}-u_{3}-\frac{\omega C_{1}^{*}}{s_{4}^{2}} & 0 & 0 & 0 & 0 \\ \displaystyle \frac{g_{2}}{s_{2}^{2}} & -\beta _{2}-d_{1} & 0 & 0 & 0\\ \displaystyle \frac{g_{3}}{s_{3}^{2}} & 0 & -\beta _{3}-d_{2} & 0 & 0\\ \displaystyle 0 & 0 & g_{4} & -\beta _{4}-d_{3}+u_{2}+\frac{\delta P_{1}^{*}}{b+P_{1}^{*}} & \frac{\delta \displaystyle C_{1}^{*}(1-P_{1}^{*})}{(b+P_{1}^{*})^{2}}\\ 0 & 0 & 0 & 0 & -\vartheta \end{array} \right) , \end{aligned}$$

and the corresponding characteristic roots are specifically

28 $$\begin{aligned} \begin{array}{llll} \displaystyle \lambda _{1}=-\beta _{2}-d_{1}<0,\quad \lambda _{2}=-\beta _{3}-d_{2}<0,\quad \displaystyle \lambda _{3}=-\vartheta <0,\\ \displaystyle \lambda _{4}=g_{1}-u_{3}-\frac{\omega C_{1}^{*}}{s_{4}^{2}},\quad \displaystyle \lambda _{5}=-\beta _{4}-d_{3}+u_{2}+\frac{\delta P_{1}^{*}}{b+P_{1}^{*}}. \end{array} \end{aligned}$$

### Theorem 4

If the characteristic roots $\lambda _{4}$ and $\lambda _{5}$ are both negative, that is, $\lambda _{4}<0$ and $\lambda _{5}<0$, then the local asymptotic stability of the equilibrium $E_{1}^{*}$ can be determined.

(ii) The Jacobian matrix of system (2) at equilibrium $E_{2}^{*}$ is

29 $$\begin{aligned} J(E_{2}^{*})= \left( \begin{array}{cccccc} \displaystyle A_{1}& A_{2} & 0 & A_{3} & 0 \\ \displaystyle A_{4} & A_{5} & 0 & 0 & 0\\ \displaystyle A_{6} & 0 & A_{7} & 0 & 0\\ \displaystyle 0 & 0 & A_{8} & A_{9} & A_{10}\\ \displaystyle 0 & 0 & 0 & 0 & -\vartheta \end{array} \right) , \end{aligned}$$

where

$$\begin{aligned} \displaystyle A_{1}&=g_{1}-u_{3}-\frac{2g_{1}T_{2}^{*}}{s_{1}}-\frac{\beta _{1}M_{2}^{*}(1-T_{2}^{*})}{(s_{2}+T_{2}^{*})^{2}}-\frac{\omega \displaystyle C_{2}^{*}(1-T_{2}^{*})}{(s_{4}+T_{2}^{*})^{2}},\\ \displaystyle A_{2}&=-\frac{\beta _{1}T_{2}^{*}}{s_{2}+T_{2}^{*}}<0,\quad \displaystyle A_{3}=-\frac{\omega T_{2}^{*}}{s_{4}+T_{2}^{*}}<0,\\ \displaystyle A_{4}&=\frac{g_{2}(1-T_{2}^{*})}{(s_{2}+T_{2}^{*})^{2}},\quad \displaystyle A_{5}=-\beta _{2}-d_{1}<0,\\ \displaystyle A_{6}&=\frac{g_{3}(1-T_{2}^{*})}{(s_{3}+T_{2}^{*})^{2}},\quad \displaystyle A_{7}=-\beta _{3}-d_{2}<0,\quad \displaystyle A_{8}=g_{4}>0,\\ \displaystyle A_{9}&=-\beta _{4}-d_{3}+u_{2}+\frac{\delta P_{2}^{*}}{b+P_{2}^{*}},\quad \displaystyle A_{10}=\frac{\delta C_{2}^{*}(1-P_{2}^{*})}{(b+P_{2}^{*})^{2}}. \end{aligned}$$

One of the characteristic roots is $\lambda _1=-\vartheta$, and the other four characteristic roots are determined by following equation

30 $$\begin{aligned} \begin{array}{llll} \lambda ^{4}-R_{1}\lambda ^{3}+R_{2}\lambda ^{2}+R_{3}\lambda +R_{4}=0, \end{array} \end{aligned}$$

where

$$\begin{aligned} R_{1}&=A_{1}+A_{5}+A_{7}+A_{9},\\ R_{2}&=A_{1}A_{5}+A_{7}A_{1}+A_{7}A_{5}+A_{9}A_{1}+A_{9}A_{5}+A_{7}A_{9}-A_{2}A_{4},\\ R_{3}&=-A_{7}A_{1}A_{5}-A_{9}A_{1}A_{5}-A_{7}A_{9}A_{1}-A_{7}A_{9}A_{5}+A_{2}A_{4}(A_{7}+A_{9})-A_{3}A_{6}A_{8},\\ R_{4}&=A_{7}A_{9}A_{1}A_{5}-A_{2}A_{4}A_{7}A_{9}+A_{3}A_{6}A_{8}A_{5}. \end{aligned}$$

Therefore, the Hurwitz matrix is:

31 $$\begin{aligned} H= \left( \begin{array}{cccccc} \displaystyle -R_{1} & R_{3} & 0 & 0 \\ \displaystyle 1 & R_{2} & R_{4} & 0\\ \displaystyle 0 & -R_{1} & R_{3} & 0 \\ \displaystyle 0 & 1 & R_{2} & R_{4} \end{array} \right) . \end{aligned}$$

According to the Routh-Hurwitz criterion, we have

32 $$\begin{aligned} \begin{array}{cccccc} H_{1}=-R_{1}>0 \Rightarrow R_{1}<0, \end{array} \end{aligned}$$

33 $$\begin{aligned} H_{2}= & \left| \begin{array}{cccccc} -R_{1} & R_{3}\\ 1 & R_{2} \end{array} \right|>0 \Rightarrow -R_{1}R_{2}-R_{3}>0, \end{aligned}$$

34 $$\begin{aligned} H_{3}= & \left| \begin{array}{cccccc} -R_{1} & R_{3}& 0\\ 1 & R_{2}& R_{4}\\ 0& -R_{1} & R_{3} \end{array} \right|>0 \Rightarrow -R_{1}R_{2}R_{3}-R_{1}^{2}R_{4}-R_{3}^{2}>0, \end{aligned}$$

35 $$\begin{aligned} \begin{array}{cccccc} H_{4}= & \textrm{det}(H)=R_{4}H_{3}>0 \Rightarrow R_{4}>0. \end{array} \end{aligned}$$

### Theorem 5

If the conditions (32)-(35) are satisfied, then the equilibrium $E_{2}^{*}$ is locally asymptotically stable.

## Examples and numerical simulations

**Application 1.** Fix the following parameter values: $Y_{0}=[10, 0, 0, 0, 0]$, $g_{1}=0.1$, $s_{1}=1000$, $s_{2}=50$, $s_{3}=50$, $s_{4}=50$,$r_{1}=0.3$, $r_{2}=0.7$, $\beta _{1}=0.1$, $\omega =0.3$, $\beta _{2}=0.5$, $g_{3}=0.3$, $\beta _{3}=0.4$, $g_{4}=0.1$, $\beta _{4}=0.7$, $d_{1}=0.008$, $d_{2}=0.0008$, $d_{3}=0.0001$, $\vartheta =1$, $\delta =0.15$, $b=0.25$.

**Fig. 1:**
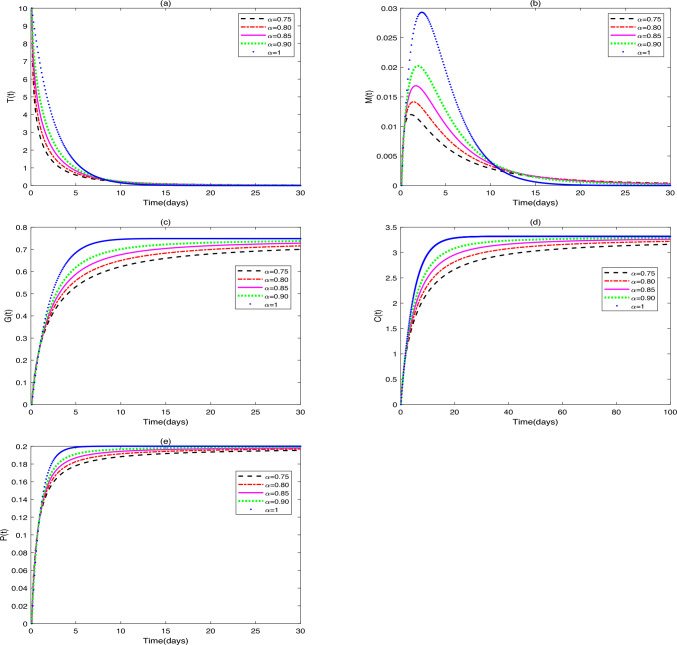
(**a**)-(**e**) are the time series of system (2) with different values of $\alpha$.

**Fig. 2:**
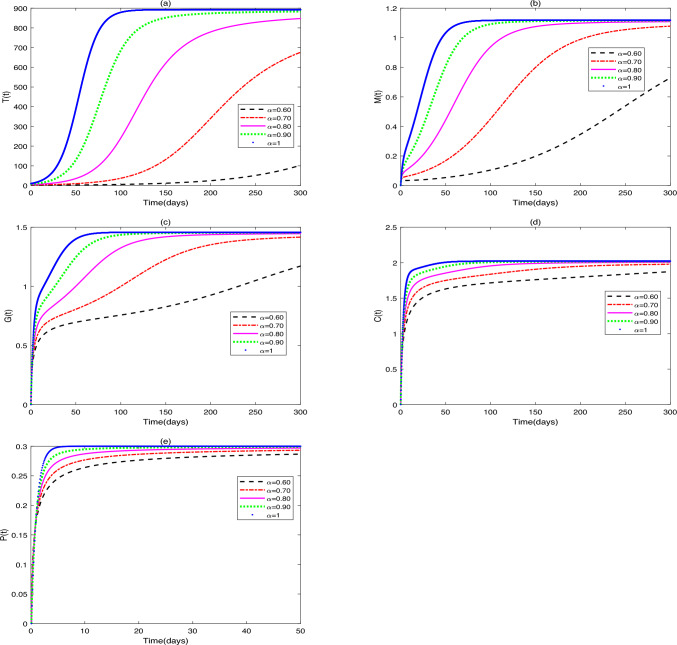
(**a**)-(**e**) are the time series of system (2) with different values of $\alpha$.

(i) Figure 1 shows the stability characteristics of equilibrium $E_{1}^{*}$ under the condition that parameters $g_{2}=0.2$ and $u_{1}=0.2$, $u_{2}=0.4$, $u_{3}=0.5$ are fixed, and meanwhile presents the influence of changes in parameter $\alpha$ $(\alpha =0.75, 0.80, 0.85, 0.90, 1)$ on the dynamic behavior of system (2).
(ii) Figure 2 shows the stability characteristics of equilibrium $E_{2}^{*}$ under the condition that parameters $g_{2}=0.6$ and $u_{1}=0.3$, $u_{2}=0.2$, $u_{3}=0.01$ are fixed, and meanwhile presents the influence of changes in parameter $\alpha$ $(\alpha =0.60, 0.70, 0.80, 0.90, 1)$ on the dynamic behavior of system (2).

**Application 2.** Fix the following parameter values: $g_{1}=0.1$, $s_{1}=1000$, $s_{2}=50$, $s_{3}=50$, $s_{4}=50$, $r_{1}=0.3$, $r_{2}=0.7$, $\beta _{1}=0.1$, $\omega =0.3$, $g_{2}=0.2$, $\beta _{2}=0.5$, $g_{3}=0.3$, $\beta _{3}=0.4$, $g_{4}=0.1$, $\beta _{4}=0.7$, $d_{1}=0.008$, $d_{2}=0.0008$, $d_{3}=0.0001$, $\vartheta =1$, $\delta =0.15$, $b=0.25$.

**Fig. 3:**
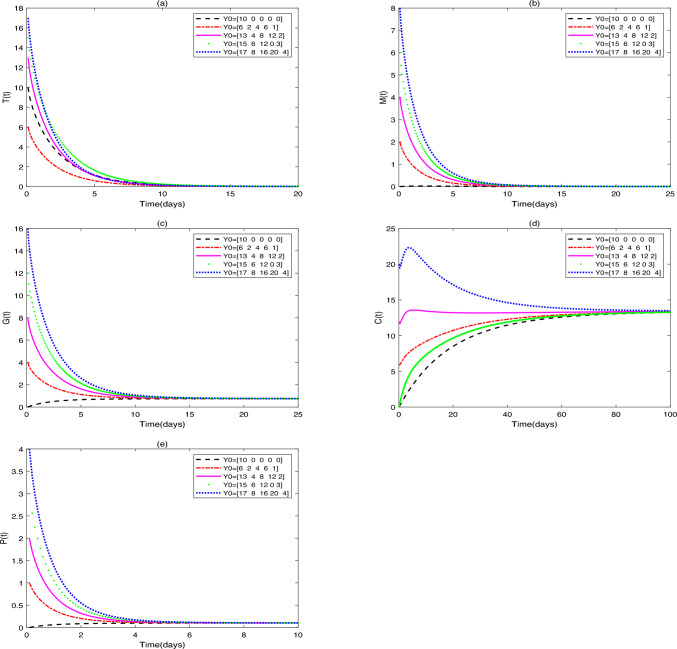
(**a**)-(**e**) are the time series of system (2) with different initial values.

**Fig. 4:**
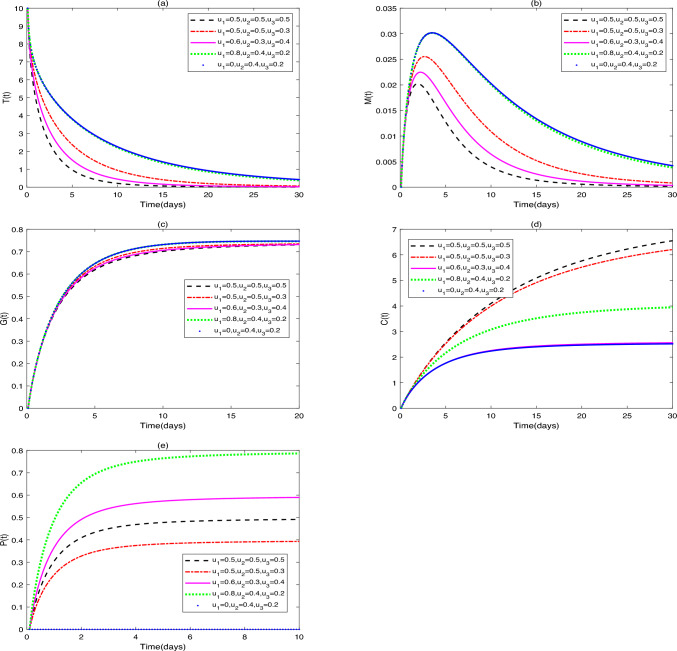
(**a**)-(**e**) are the time series of system (2) with different values of $u_{1}$, $u_{2}$, $u_{3}$.

(i) In Fig. 3, fix $\alpha =0.96$ and $u_{1}=0.1$, $u_{2}=0.6$, $u_{3}=0.5$. Figure 3 shows the influence of initial values $(Y_{0}=[10, 0, 0, 0, 0]$, [6, 2, 4, 6, 1], [13, 4, 8, 12, 2], [15, 6, 12, 0, 3], [17, 8, 16, 20, 4]) on the dynamic behavior of system (2).
(ii) In Fig. 4, fix $\alpha =0.90$ and initial values $Y_{0}=[10, 0, 0, 0, 0]$. Figure 4 demonstrates the influence of different drug dosages $([u_{1}, u_{2}, u_{3}]=[0.5, 0.5, 0.5]$, [0.4, 0.5, 0.3], [0.6, 0.3, 0.4], [0.2, 0.4, 0.2], [0.1, 0.2, 0.1]) on the dynamic behavior of system (2).

However, in the medical field, there are more interactions among various cells. Based on this, we will further expand the application scope of the model and construct the following generalized model:

36 $$\begin{aligned} \left\{ \begin{array}{lll} \displaystyle ^{C}_{0}D_{t}^{\alpha }T(t)=g_{1}T(t)\left( 1 -\frac{T(t)}{s_{1}}\right) -\beta _{1}\frac{T(t)M(t)}{s_{2}+T(t)}-\omega \frac{T(t)C(t)}{s_{4}+T(t)}-u_{3}(t)T(t),\\ \displaystyle ^{C}_{0}D_{t}^{\alpha }M(t)=g_{2}\frac{T(t)}{s_{2}+T(t)}-\beta _{2}M(t)-d_{1}M(t)+g_{5}\frac{C(t)}{s_{2}+C(t)},\\ \displaystyle ^{C}_{0}D_{t}^{\alpha }G(t)=r_{1}+g_{3}\frac{T(t)}{s_{3}+T(t)}-\beta _{3}G(t)-d_{2}G(t) +g_{6}\frac{M(t)}{s_{3}+M(t)}+g_{7}\frac{C(t)}{s_{3}+C(t)},\\ \displaystyle ^{C}_{0}D_{t}^{\alpha }C(t)=r_{2}+g_{4}G(t)-\beta _{4}C(t)-d_{3}C(t)+\frac{\delta P(t)C(t)}{b+P(t)}+u_{2}(t)C(t),\\ \displaystyle ^{C}_{0}D_{t}^{\alpha }P(t)=u_{1}(t)-\vartheta P(t), \end{array}\right. \end{aligned}$$

In this model, $g_{5}$, $g_{6}$, and $g_{7}$ respectively characterize the recruitment rates of macrophages by CD4+T helper cells, dendritic cells by macrophages, and dendritic cells by CD4+T helper cells. We study the following application:

**Application 3.** Fix the following parameter values: $Y_{0}=[10, 0, 0, 0, 0]$, $s_{1}=1000$, $s_{2}=50$, $s_{3}=50$, $s_{4}=50$, $r_{1}=0.3$, $r_{2}=0.7$, $\beta _{1}=0.1$, $\omega =0.3$, $g_{2}=0.2$, $\beta _{2}=0.5$, $g_{3}=0.3$, $\beta _{3}=0.4$, $g_{4}=0.1$, $\beta _{4}=0.7$, $d_{1}=0.008$, $d_{2}=0.0008$, $d_{3}=0.0001$, $\vartheta =1$, $\delta =0.15$, $b=0.25$, $\alpha =0.90$, $u_{1}=0.2$, $u_{2}=0.4$, $u_{3}=0.5$, $g_{5}=0.1$, $g_{6}=0.15$, $g_{7}=0.2$.

**Fig. 5:**
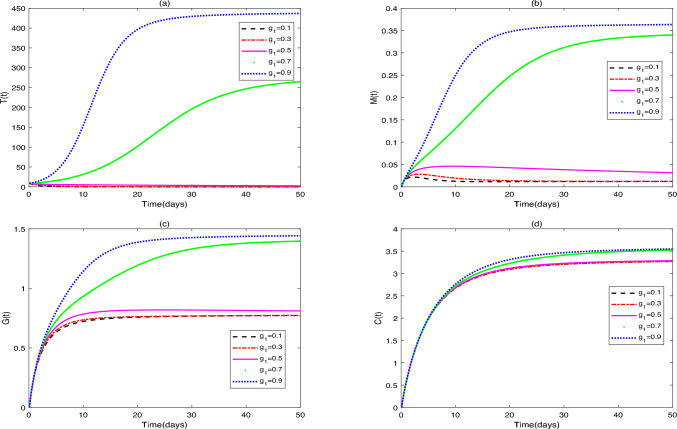
(**a**)-(**d**) are the time series of system (36) with different values of $g_{1}$.

**Fig. 6:**
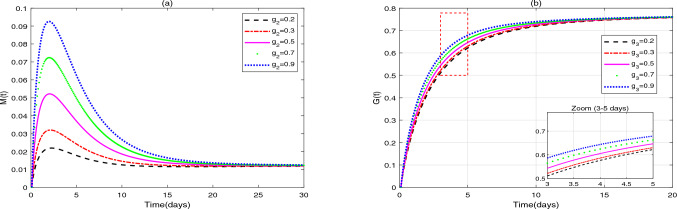
(**a**), (**b**) aer the time series of system (36) with different values of $g_{2}$, $g_{3}$.

**Fig. 7:**
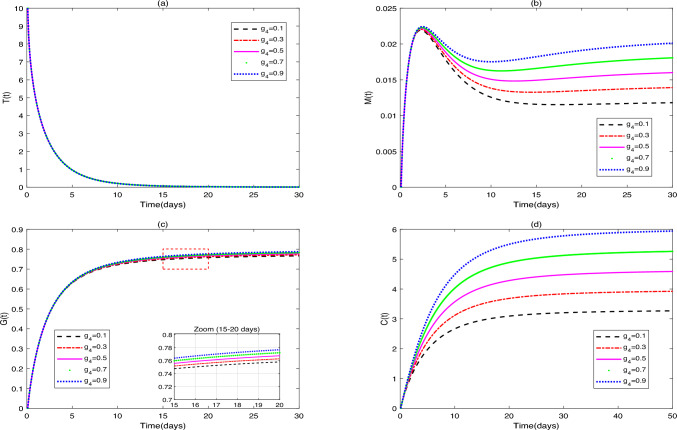
(**a**)-(**d**) are the time series of system (36) with different values of $g_{4}$.

**Fig. 8:**
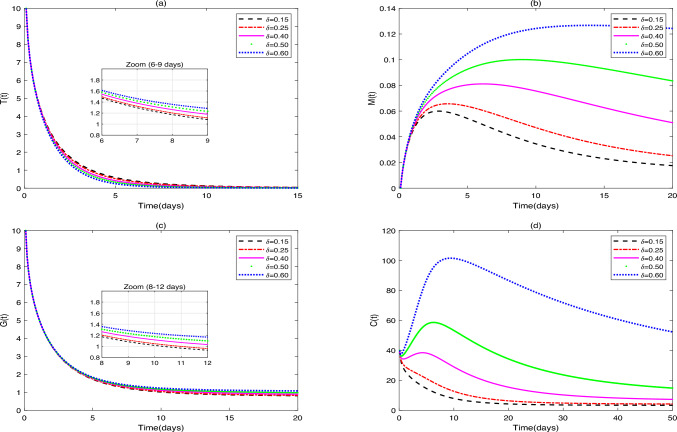
(**a**)-(**e**) are the time series of system (36) with different values of $\delta$.

(i) Figures 5, 6, 7, 8 show the influence of $g_1$, $g_2$, $g_3$, $g_4$, and $\delta$ on the dynamic behavior of system (36), fix the parameter values $g_{1}=[0.1, 0.3, 0.5, 0.7, 0.9]$, $g_{2}=[0.2, 0.3, 0.5, 0.7, 0.9]$, $g_{3}=[0.2, 0.3, 0.5, 0.7, 0.9]$, $g_{4}=[0.1, 0.3, 0.5, 0.7, 0.9]$, $\delta =[0.15, 0.25, 0.40, 0.50, 0.60]$. Additionally, the initial value in 8 is set as $Y_{0}=[10, 0, 10, 40, 40]$.

### Remark 1

(i) It can be seen from Figs. 1 and 2 that does not affect the stability of the equilibriums, but influences the speed of approaching equilibrium.
(ii) It can be seen from Fig. 3 that the initial values do not affect the stability of the equilibriums, but have an impact on the speed of approaching equilibrium.
(iii) As can be seen from Fig. 4, when patients receive different drug dosages, there are differences in the treatment effects obtained.

## Discussions and conclusions

While probiotic antitumor mechanisms often focus on remodeling the TME and modulating T cell infiltration, existing research prioritizes $\hbox {CD8}^{+}$T cell cytotoxicity over $\hbox {CD4}^{+}$T cell immunoregulatory roles. This study therefore focuses on $\hbox {CD4}^{+}$T cells for in-depth analysis. As a key T cell subset expressing CD4, $\hbox {CD4}^{+}$T cells serve as the foundation for immunotherapy and undergo complex functional/phenotypic remodeling during chemotherapy.

The role of $\hbox {CD4}^{+}$T cells is to orchestrate adaptive immunity not through direct cytotoxicity, but by secreting cytokines and assisting other immune cells. This function includes activating $\hbox {CD8}^{+}$T cell proliferation/differentiation, promoting B cell antibody production, and regulating macrophages and dendritic cells (DCs). Prognostic signatures associated with $\hbox {CD4}^{+}$T cells signatures predict outcomes and immunotherapy responses in patients with stage III-IV CRC^30^.

In this study, Figs. 1, 2 illustrate that differences in the values of $\alpha$ affect the speed at which the equilibriums $E_{1}$ and $E_{2}$ tend to stability. The larger the value of $\alpha$, the faster the growth rate of the numbers of tumor cells, macrophages, dendritic cells and $\hbox {CD4}^{+}$T helper cells, and the higher the growth rate of probiotic dosage. Variations in the fractional-order parameter $\alpha$ primarily affect the rate at which the system approaches equilibrium, reflecting the “time-memory effect” in biological systems. Biologically, $\alpha$ quantifies the timescale of intercellular interactions: immune cell activation, proliferation, and drug metabolism in the TME exhibit temporal accumulation. At low $\alpha$ values (e.g., $\alpha$ = 0.75), macrophage and dendritic cell numbers increase slowly, mirroring delayed immune activation in chronic inflammation, where sustained cytokine release requires time to trigger effective immune cascades. Studies confirm that immunosuppressive function of tumor-infiltrating Tregs ($FoxP3^{+}$) peaks 48–72 hours post-inflammatory stimulation in CRC patients, preceding effector T cell activation (96 hours)^31^. At high $\alpha$ (e.g., $\alpha$ = 1), tumor cells display rapid exponential growth before stabilizing, aligning with the Gompertz model’s “early unrestricted proliferation followed by constrained regression” in solid tumors. Model predictions corroborate experimental data: Suzuki quantified tumor cell growth in HCT-116-luc2 models via luciferase intensity, with time-intensity curves fitting exponential functions^32^. Higher $\alpha$ accelerates initial tumor proliferation but also hastens immune cell/probiotic activation, ultimately expediting tumor clearance.

Probiotic concentration increases monotonically to equilibrium, indicating sustained colonization and metabolic activity in vivo. Probiotic effects depend on time-dependent epithelial adhesion and require a stabilization period. Li et al. observed peak colonization of *Dsred2*-labeled *L. rhamnosus* in BALB/c mice on day 4 post-gavage^33^. Under triple-drug exposure (Fig. 1), macrophages exhibit a bell-shaped curve: initial recruitment by tumor cells increases their numbers, followed by decline due to chemotherapy-induced damage and natural death. This likely reflects macrophages’ dual role in the TME: early tumor recruitment (via CSF-1 etc.) promotes M2 polarization and immunosuppression; chemoimmunotherapy reverses polarization (toward M1) but chemotherapy toxicity reduces survival, causing late-phase depletion.

Figure 3 demonstrates that the dynamic system governed by our model exhibits global stability, a key theoretical insight. While varying initial conditions-simulating significant interpatient heterogeneity in baseline tumor burden and immune status-affect the rate at which the system approaches equilibrium, they do not alter the final stability outcome. This indicates that the therapeutic strategies modeled (chemo-immunotherapy combined with probiotics) are robust across a diverse patient population, consistently driving the system toward a predictable steady state (tumor clearance or coexistence). A detailed analysis of the trajectories reveals the critical role of specific initial immune states: High initial immune cell counts, particularly of $\hbox {CD4}^{+}$T cells, function as a pre-existing immune advantage, significantly accelerating the trajectory toward tumor clearance. This mathematically corroborates clinical observations where the quantification of 22 immune cell types in colorectal cancer (CRC) transcriptomes revealed that $\hbox {CD4}^{+}$ memory-activated T cell infiltration is a positive prognostic marker, correlating with longer relapse-free survival^34^. Conversely, the absence of initial $\hbox {CD4}^{+}$T cells delays tumor clearance, as the system must first undertake a time-consuming immune reconstitution process. This process relies on dendritic cell (DC) activation and, crucially, on probiotic-driven recruitment and polarization of $\hbox {CD4}^{+}$T cells from scratch, as captured by the model term $\delta P(t)C(t)/(b+P(t))$. These simulation results directly inform potential clinical translation. They suggest that probiotic loading doses could be a strategic intervention to rapidly elevate $\hbox {CD4}^{+}$T cell levels *C*(*t*) at the treatment outset, thereby optimizing early efficacy. This is supported by findings that Lactobacillus rhamnosus GG extracellular vesicles (LGG-EVs) can rapidly modulate intestinal immunity by boosting populations of MHC II$^{+}$ DCs, $\hbox {CD4}^{+}$T, and $\hbox {CD8}^{+}$T cells in tumors^35^. Consequently, our model predicts that patients with a high initial tumor burden or an immunocompromised state may benefit from higher probiotic induction doses to achieve faster tumor control, underscoring the potential of early probiotic intervention to reshape a patient’s initial immune status for improved therapeutic outcomes.

Figure 4 explores dose-dependent dynamics. Probiotic monotherapy shows limited efficacy, necessitating combination regimens. Full-dose triple therapy shortens tumor clearance time versus mono/dual therapies. Augmenting probiotics alongside fixed chemoimmunotherapy doses significantly increases $\hbox {CD4}^{+}$T cell recruitment rates, aligning with clinical data: a meta-analysis of 6 RCTs involving 492 patients with perioperative CRC found that enteral probiotics elevated serum IgG/IgA/IgM, $\hbox {CD4}^{+}$T cells, and $\hbox {CD4}^{+}$T/CD8$^{+}$T ratios^36^. However, tumor clearance and macrophage/DC recruitment rates remain similar. We posit that despite increasing $\hbox {CD4}^{+}$T cell numbers, insufficient chemo/immunotherapy dosing or excessive macrophage damage may prevent single immune cell augmentation from overcoming tumor immune escape. It can be postulated that despite the increase in $\hbox {CD4}^{+}$T cell numbers, insufficient chemo/immunotherapy dosing or excessive macrophage damage might prevent single immune cell augmentation from overcoming tumor immune escape.

Functionally, $\hbox {CD4}^{+}$T cells comprise helper T (Th) and regulatory T (Treg) subsets. Th1 cells exert antitumor effects: *Akkermansia muciniphila* supplementation promotes Th1 responses, increases $\hbox {CCR9}^{+}$CXC- R3$^{+}$ $\hbox {CD4}^{+}$T cell recruitment to tumors in an IL-12-dependent manner, and restores PD-1 blockade efficacy. *A. muciniphila* $+$ anti-PD-1 reduced tumor volume by 68% versus anti-PD-1 alone ($p<0.001$), particularly in MSS tumors^37^. Conversely, Th17 cells correlate positively with colorectal inflammation and tumorigenesis. Fecal microbiota transplantation from CRC patients to germ-free/conventional mice elevated proinflammatory cytokines, increased intestinal polyps, and expanded Th17 cells, accelerating CRC progression^38^. T follicular helper (Tfh) cells, essential for germinal center (GC) formation, support B cells and antibody production^39^. *Helicobacter hepaticus* colonization induces microbiota-specific Tfh cells, promoting tertiary lymphoid structure (TLS) formation and antitumor immunity^40^. Tregs typically suppress antitumor immunity. *L. gallinarum* synergizes with anti-PD1 therapy by suppressing intratumoral Foxp3$^{+}$CD25$^{+}$ Tregs and boosting $\hbox {CD8}^{+}$T cell effector function^41^. Additionally, the novel IL-9-secreting Th9 subset exhibits paradoxical roles in tumor development, with both pro-and antitumor activities reported^42^.

It was noted that immune cells exhibited differential drug sensitivity: macrophages were chemotherapy-sensitive (with lower peaks at $u_{3}=0.5$), in contrast to dendritic cells (DCs), whose activation was more reliant on immunotherapy dosage (with faster activation at $u_{2}=0.6$). This aligns with Fauvre et al.: flow cytometry analysis revealed oxaliplatin recruited neutrophils and macrophages via Ccl2/Cxcl1 upregulation, whereas anti-PD-1 increased $\hbox {CD8}^{+}$T cell infiltration but minimally suppressed neutrophils or M2 macrophages^43^. Although mechanisms require further exploration, these differences highlight cell-type-specific treatment responses in the TME, underscoring the complementary mechanisms of chemo-and immunotherapy.

Figure 5 identifies growth rate ($g_{1}$) as a key driver of tumor burden. Macrophage and DC numbers rise with increasing *g*1, likely due to tumor-driven immune stimulation. $\hbox {CD4}^{+}$T cell levels, however, are modulated by *g*4 and immunotherapy dose ($u_{2}$), showing gentler trends.

Figure 6 shows macrophage enrichment directly correlates with tumor recruitment capacity ($g_{2}$). Tumor-associated macrophages (TAMs) exhibit high plasticity, polarizing into proinflammatory M1 or protumor M2 phenotypes. With tumor progression, the initially antitumoral M1-like TAMs undergo a shift toward the M2 phenotype, thereby promoting tumorigenesis^44^. Macrophages also influence other immune cells: IL-12/IFN-$\gamma$ secretion activates and matures DCs, facilitating their migration to tumors. Direct contact enables antigen transfer from macrophages to DCs for subsequent T cell presentation^45^.

As APCs, DCs are pivotal in adaptive immunity. High DC infiltration correlates with favorable CRC prognosis^46^. DC-based vaccines are among the most widely used cancer immunotherapies^47^. Yan et al. designed a liposomal nano vaccine that, combined with immunotherapy, enhances DC antigen presentation and tumor-specific T cell activation^48^. DC interactions within the TME critically influence tumor progression. Tumor-derived extracellular vesicles containing miR-424 inhibit CD28-CD80/86 costimulation in T cells and DCs, conferring resistance to immune checkpoint blockade^49^. Combining oxaliplatin, the antiangiogenic agent fruquintinib, and anti-PD-1 increases cytotoxic T cells, DCs, and NK cells in the TME, suppressing colon tumor growth^50^. $\hbox {CD4}^{+}$T cell functionality relies on dynamic crosstalk with other immune cells.

In Fig. 7, with the increase in the value of parameter $g_{4}$, the numbers of tumor cells, macrophages, and dendritic cells all show an increasing trend. Satoru et al. observed CD68$^{+}$ macrophages co-localized with Th1 cells; Th1-derived cytokines polarize macrophages toward M1, enhancing phagocytosis and tumor killing. DCs uptake tumor antigens in TLSs, migrate to lymph nodes, and prime naïve CD$4^{+}$T cells for Th1 differentiation^51^. Under chemoimmunotherapy, intratumoral Tregs convert to Th1 effectors, TAMs are depleted, and cytotoxic T lymphocyte infiltration/activation increases^52^. High Th17 cell levels and *IL17A/Rorc* expression correlate with poor CRC prognosis. Beyond T cells, DCs and macrophages secrete IL-17A to suppress Th1/Th2/Th17 development and inflammation^53^. Collectively, $\hbox {CD4}^{+}$T cells, $\hbox {CD8}^{+}$T cells, macrophages, and DCs form a complex immunoregulatory network, where dynamic interactions and functional synergy drive antitumor immunity.

Probiotic antitumor mechanisms are rooted in precisely modulating this immune network. By remodeling the gut microbiome or directly targeting the TME, probiotics recruit $\hbox {CD4}^{+}$T cells and polarize them toward effector subsets (e.g., Th1), using $\hbox {CD4}^{+}$T cells as “signaling hub” to activate cascading immune responses.

Although the parameter $g_{4}$ has different values, the time evolution curve of tumor cells shows high consistency.In summary, on one hand, changes in $g_{4}$ only indirectly affect tumor cells by modulating the activation of $\hbox {CD4}^{+}$T cells *C*(*t*) by dendritic cells *G*(*t*). The sensitivity to variations in $g_{4}$ is determined by the coupling strength of this indirect pathway. On the other hand, under the given parameter set (particularly with the chemotherapy dose $u_{3}=0.5$), the direct cytotoxic effect of the drug likely dominates the tumor regression dynamics, thereby diminishing the relative contribution of immunomodulation to the overall tumor behavior. These findings suggest that in the context of intensive chemotherapy, merely enhancing the dendritic cell-$\hbox {CD4}^{+}$T cell axis may be insufficient to further improve tumor clearance. Consequently, the $\hbox {CD4}^{+}$T cell activation rate $g_{4}$ does not serve as a primary regulatory factor in tumor dynamics.

In Fig. 8, increasing probiotic-mediated $\hbox {CD4}^{+}$T cell recruitment elevates macrophage and DC numbers. This observation is mechanistically explained by the nonlinear growth term $\delta P(t)C(t)/(b+P(t))$ in the dynamic equation of $\hbox {CD4}^{+}$T cells (*C*(*t*)). An increase in $\delta$ signifies that probiotics can more efficiently recruit and polarize existing T cells. This “amplifier” effect enhances the output of the entire immune network more directly and markedly, resulting in the significant increase in all immune cells and accelerated tumor clearance. This modeled mechanism is consistent with established biology: activated $\hbox {CD4}^{+}$T cells secrete cytokines (e.g., IFN-$\gamma$) to promote DC maturation, drive macrophage M1 polarization, and recruit cytotoxic T lymphocytes. This $\hbox {CD4}^{+}$T cell-centric network remodeling allows probiotics to disrupt the immunosuppressive TME holistically. This is supported by studies showing that probiotics enhance anti-PD-1 efficacy via $\hbox {CD4}^{+}$T cell activation and DC cross-presentation^54^, and that probiotic OMVs promote M1 polarization and DC maturation^55^. Probiotics also alleviate chemotherapy-induced mucositis in CRC rats by modulating T cell subsets: *B. infantis* reduced $\hbox {CD4}^{+}$IL-17A$^{+}$ cells and increased $\hbox {CD4}^{+}$CD25$^{+}$Foxp3$^{+}$ Tregs in mesenteric lymph nodes versus chemotherapy alone^56^. Further studies are needed to validate probiotics’ role in mitigating chemoimmunotherapy toxicity via T cell modulation.

This study provides a mathematical framework for modeling CRC immune dynamics and elucidates probiotics’ pivotal role in cancer therapy. Future work should integrate single-cell sequencing and immune monitoring to refine model parameters, advancing fractional-order models from theoretical tools to clinical predictors. There are still some limitations. The model focuses solely on $\hbox {CD4}^{+}$T cells, omitting $\hbox {CD8}^{+}$T cells; a globally uniform $\alpha$ value is assumed, whereas biological components may have distinct timescales; spatial tumor distribution (e.g., necrotic core, proliferative edge) and immune infiltration heterogeneity are neglected, potentially inaccurately depicting drug diffusion and cell migration.

As a simplified mathematical simulation, the model cannot fully capture the complexity of in vivo immune networks. Future studies will develop more sophisticated models integrating multidimensional biological data and dynamic parameters for precise mechanistic exploration.

## Data Availability

Data are contained within the article.
